# Screening of temperature-responsive signalling molecules during sex differentiation in Asian yellow pond turtle (*Mauremys mutica*)

**DOI:** 10.1186/s12864-024-10275-5

**Published:** 2024-04-18

**Authors:** Xiaoli Liu, Haoyang Xu, Mingwei Peng, Chenyao Zhou, Chengqing Wei, Xiaoyou Hong, Wei Li, Chen Chen, Liqin Ji, Xinping Zhu

**Affiliations:** 1 Key Laboratory of Tropical and Subtropical Fishery Resources Application and Cultivation, Ministry of Agriculture and Rural Affairs, Pearl River Fisheries Research Institute, Chinese Academy of Fishery Sciences, 510380 Guangzhou, China; 2 College of Life Science and Fisheries, Shanghai Ocean University, 201306 Shanghai, China; 3 School of Fishery, Zhejiang Ocean University, 316000 Zhoushan, China

**Keywords:** Turtle, Gonadal transcriptome, RNA-seq, Sexual dimorphism, Temperature-dependent sex determination

## Abstract

**Background:**

The Asian yellow pond turtle (*Mauremys mutica*) is an important commercial freshwater aquaculture species in China. This species is a highly sexually dimorphic species, with males growing at a faster rate than females and exhibits temperature-dependent sex determination (TSD), in which the incubation temperature during embryonic development determines the sexual fate. However, the mechanisms of the sex determination or sex differentiation in the Asian yellow pond turtle are remain a mystery.

**Results:**

Temperature-specific gonadal transcriptomics of the Asian yellow pond turtle were performed during the thermosensitive period (stage 15) using RNA-seq technology to identify candidate genes that initiate gonadal differentiation. We uncovered candidates that were the first to respond to temperature. These candidates were sexually dimorphic in expression, reflecting differences in gonadal (*Cirbp*, *Runx1*) and germline differentiation (*Vasa*, *Nanos1*, *Piwil2*), gametogenesis (*Hmgb3*, *Zar1*, *Ovoinhibitor-like*, *Kif4*), steroid hormone biosynthesis (*Hsd17b5*, *Hsd17b6*), heat shock (*Dnajb6*, *Hsp90b1*, *Hsp90aa1*) and transient receptor potential channel genes (*Trpm1*, *Trpm4*, *Trpm6*, *Trpv1*).

**Conclusions:**

Our work will provide important genetic information to elucidate the mechanisms of sex control in the Asian yellow pond turtles, and will contribute important genetic resources for further studies of temperature-dependent sex determination in turtles.

**Supplementary Information:**

The online version contains supplementary material available at 10.1186/s12864-024-10275-5.

## Background

Reptiles exhibit a diverse range of sex determination mechanisms and plastic patterns of sex differentiation among amniote vertebrates [1]. Their sexual phenotype can be determined by genotype or environment, referred to as genotypic sex determination (GSD) and environmental sex determination (ESD), respectively [2]. Recent studies have demonstrated that a single species can exhibit both genotypic and environmental effects (GSD + ESD) [3, 4]. Among several environmental factors such as temperature [5], photoperiod [6], social factors [7] and dissolved oxygen (DO) [8], incubation temperature is the only identified element that influences sex determination and triggers gonadal differentiation into ovaries or testes in reptiles and is thus referred to as temperature-dependent sex determination (TSD). TSD can be divided into three main patterns based on the sex ratio of offspring produced at different temperatures: Ia (also known as the MF pattern), Ib (also known as the FM pattern) and Type II (also known as the FMF pattern) [9]. The Ia pattern is found only in turtles, and shows a preference for females at high incubation temperatures and males at low temperatures [10]. The Ib pattern produces females at low temperature and males at high temperature, as in *Sphenodon punctatus* [11]. Type II TSD produces females at both low and high temperature, males at intermediate temperatures, and is found in all groups of reptiles including turtles, crocodiles, and lizards [12, 13]. Reptiles are therefore ideal models for elucidating the mechanisms of TSD.

For almost 50 years, research into the causes of variation in TSD has focused on two main questions: (1) How does the temperature signal translate into biological signals and activate key regulators of gonadal differentiation? (2) Which genes regulate dimorphic gonadal differentiation into ovaries or testes? Several key genes, including *dmrt1*, *sox9*, *amh*, and *cyp19a1*, which are relatively conserved in the GSD model, have been reported in TSD turtles. These genes can initiate dimorphic gonadal differentiation into males or females [6, 14, 15]. In TSD animals, male and female individuals have nearly identical genetic background, but there are significant differences in sex characteristics. This has led to speculation that epigenetics may play a role in regulating TSD. In *Trachemys scripta elegans* (*T*. *scripta*), signal transducer and activator of transcription 3 (STAT3) can block the male pathway by binding to the histone H3 lysine 27 (H3K27) demethylase Kdm6b locus to repress Kdm6b transcription [16]. Kdm6b exhibits temperature-dependent sexually dimorphic expression in early *T. scripta* embryos. It can directly promote the transcription of the male sex-determining gene *dmrt1* by removing the trimethylation of H3K27 near its promoter [10, 17]. This work has been highly praised in a review article published in *Science*, which believes that it solves the mystery of TSD research for half a century and is a major original breakthrough in the field of life sciences.

The Asian yellow pond turtle (*Mauremys mutica*), is a species of the Geoemydidae family, and the *Mauremys* genus. It is widely distributed in China [18], Vietnam and Japan [19]. The turtle has high edible, medicinal and ornamental value and has become an important economic aquaculture species in China [20, 21]. In 2002, it was listed in Appendix II of CITES and listed as endangered on the IUCN Red List of Threatened Species due to over-hunting and habitat destruction in the wild. Captive breeding has become an important measure to meet commercial demand and improve the conservation of the Asian yellow pond turtle [22], thanks to breakthroughs in artificial breeding techniques. The species exhibits significant sexual dimorphism, with males growing faster than females [23]. Analysing the mechanism of temperature sex determination in Asian yellow pond turtles will not only improve our understanding of the evolutionary mechanism of sex determination in turtles, but also provide theoretical support for sex control and production breeding of Asian yellow pond turtles. Similar to the inheritance or absence of the Y-linked gene *Sry* in mammals, the sex of the Asian yellow pond turtle is determined by a bimodal developmental response to egg incubation temperature with high incubation temperature producing female offspring and low incubation temperature producing male offspring [24]. Significant progress has been made in studying temperature-dependent sex determination mechanisms in *T. scripta*. However, the control of gonadal sex determination is remarkably diverse [12]. Therefore, it is crucial to identify more upstream sex-determining genes, study their position in the hierarchical network of sex control, and construct a cascade system to regulate sex development. A high-quality genome assembly of the Asian yellow pond turtle was recently generated using continuous long read (PacBio platform), Illumina and high-throughput chromatin conformation capture (Hi-C) technologies [25]. Subsequently, several sex-specific markers were screened using multi-omics conjoint analysis [18, 26]. However, the overall temporal pattern of sex-specific genes responding to temperature remains poorly understood.

In this study, the temperature-specific gonadal transcriptomics of the Asian yellow pond turtle were performed during the thermosensitive period to identify candidate genes that initiate gonadal differentiation based on RNA-seq analysis. A list of candidates that are temperature responsive and sexually dimorphic in their expression was identified. Our study aims to provide genetic information to understand the mechanisms of sex control in Asian yellow pond turtles. Additionally, it will serve as a valuable genetic resource for further research on temperature-dependent sex determination in turtles.

## Methods

### Sampling and incubation of turtle eggs

Fresh fertilised *M. mutica* eggs were obtained from the Guangzhou Aquatic Thoroughbred Base of the Pearl River Fisheries Research Institute. Normally sized eggs were collected from sand ponds within one day of spawning and then transferred to a constant humidity incubator at 25 °C (MPT) or 32 °C (FPT) for hatching. A total of 300 fertilised eggs were placed at each temperature. In *M. mutica*, the gonads can be separated from the mesonephric complex under the microscope at stage 15. To investigate the effect of temperature on the embryonic development, temperature shift experiments were performed before stage 15. A total of 150 *M. mutica* eggs at stage 15 were transferred from an incubator at 25 °C to an incubator at 32 °C and vice versa. Embryos at 6, 12, 24, 48 and 72 h after temperature shift, as well as embryos at corresponding time points without a temperature shift, were dissected and placed in PBS. Embryonic gonads were separated and collected, and 10 embryos with 20 gonads were pooled as one sample. Two biological duplicates of each stage and temperature were collected. The embryos were staged according to the morphological characterization established by Greenbaum [27] and Zhao [28]. The animal experiments in this study were conducted in accordance with the guidelines of the Pearl River Fisheries Research Institute, Chinese Academy of Fishery Sciences. The turtles used were treated humanely and ethically, and the experiments were approved by the Pearl River Fisheries Research Institute, Chinese Academy of Fishery Sciences.

### RNA extraction, library construction and Illumina sequencing

Total RNA was extracted from each sample using the SV Total RNA Isolation System (Promega, USA) following the manufacturer’s protocol. RNA concentration and purity were assessed using the NanoDrop 2000 spectrophotometer (Thermo Fisher Scientific, Wilmington, DE). RNA integrity was assessed using the RNA Nano 6000 Assay Kit of the Agilent Bioanalyzer 2100 System (Agilent Technologies, CA, USA). Qualified RNA was processed for library construction. The procedures are described as follows: (1) Isolation of mRNA was carried out using oligo(dT)-attached magnetic beads. (2) The mRNA was then randomly fragmented in fragmentation buffer. (3) First-strand cDNA was synthesised using fragmented mRNA as template and random hexamers as primers, followed by second-strand synthesis with the addition of PCR buffer, dNTPs, RNase H and DNA polymerase I. Purification of cDNA was performed using AMPure XP beads. (4) The double-strand cDNA was subjected to end repair, followed by the addition of adenosine and ligation to adapters. Fragments in the size range of 300–400 bp were selected using AMPure XP beads. (5) The cDNA library was generated by several rounds of PCR on the cDNA fragments generated in step 4. To ensure library quality, the concentration of cDNA and insert size were checked using Qubit 2.0 and Agilent 2100. Q-PCR was performed to obtain a more accurate library concentration. A library with a concentration great than 2 nM is acceptable. The qualified library was pooled based on the pre-designed target data volume and then sequenced on the TruSeq PE Cluster Kit v4-cBot-HS Illumina sequencing platform.

### Quality control and assembly of the gonadal transcriptome

Using sequencing-by-synthesis (SBS) technology, cDNA libraries were sequenced on the Illumina high-throughput platform, generating significant amounts of high quality raw data stored in FASTQ format. Each sample has two FASTQ files, each containing cDNA reads measured at both ends. The raw data was processed using in-house Perl scripts to remove adapter contamination and nucleotides with low quality scores. The processed data was then converted into clean reads. The resulting clean reads were then mapped to *M. mutica* reference genome sequence (SRR14883730) using Hisat2 (http://ccb.jhu.edu/software/hisat2/index.shtml) [29]. Only reads with a perfect match or one mismatch were further analysed and annotated against the reference genome. The gene function was annotated using several databases, including Nr (NCBI non-redundant protein sequences), Nt (NCBI non-redundant nucleotide sequences), Pfam (Protein family), KOG/COG (Clusters of Orthologous Groups of proteins), Swiss-Prot (A manually annotated and reviewed protein sequence database), KO (KEGG Ortholog database), and GO (Gene Ontology).

### Identification of differentially expressed genes (DEGs)

Gene expression levels were quantified using fragments per kilobase of transcript per million fragments mapped (FPKM) [30]. Differential expression analysis of two time points was performed using the DESeq2 [31]. The resulting *P*-values were adjusted using the Benjamini and Hochberg’s approach to control for false discovery rate. Genes with an adjusted *P*-value < 0.05 identified by DESeq2 were assigned as DEGs.

## Results

### Sample collection according to the gonadal differentiation time

**Fig. 1:**
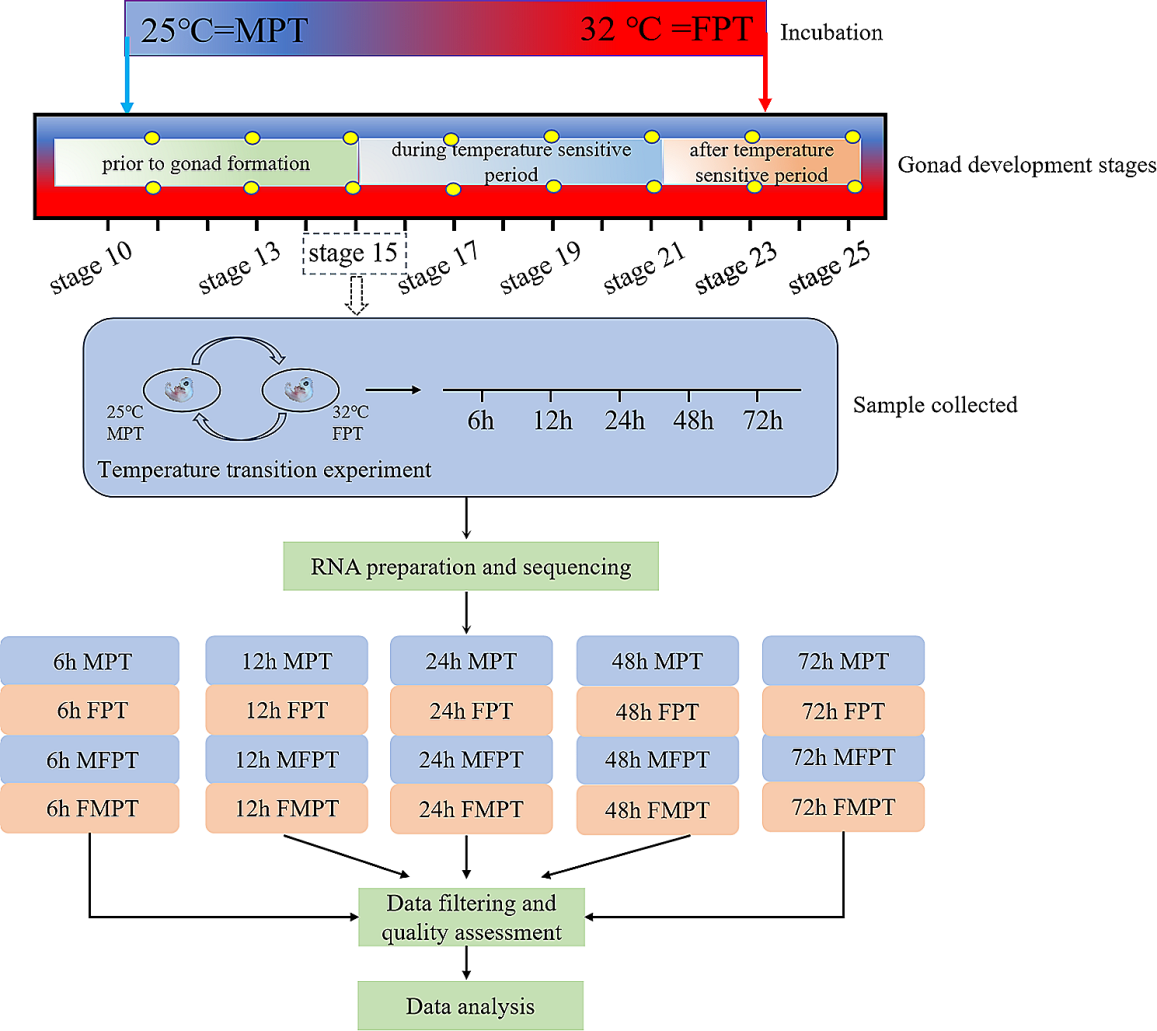
Experimental design for RNA-seq in the Asian yellow pond turtle. The incubation temperature of 25℃ was the male produce temperature (MPT), while 32℃ was the female produce temperature (FPT). Embryo transcriptomes of MPT, FPT, and temperature shifts (25 °C to 32 °C and 32 °C to 25 °C) were collected at 5 time points (6 h, 12 h, 24 h, 48 h and 72 h) in the thermosensitive period (stages 15)

Histological and expression analyses of sex-regulated genes [32] revealed no significant differences between the female and the male gonads up to the stage 15. At stage 15, the male gonads became irregularly polygonal in shape, with the cortical region degenerating into a single cell layer, the medullary region developing, and the primitive gonadal cords forming and supporting cellular precursors surrounding the primitive germ cells. The gonads of the female were umbrella-shaped, with developing cortical areas and gradually degenerating medullary areas, and a large number of germ cells distributed in the cortical areas (Fig. S1). To determine which factors respond first to changes in temperature, we sampled male and female gonads at two different temperatures: 25 °C (male produced temperature, MPT) and 32 °C (female produced temperature, FPT) of stage 15, as well as embryos at 6, 12, 24, 48 and 72 h after the temperature shift (from 25 °C to 32 °C and from 32 °C to 25 °C). We constructed a total of 40 cDNA libraries from the male and female gonads for the subsequent transcriptome sequencing (Fig. 1).

### Summary statistics of RNA‑seq data

The transcriptomes of 40 samples in four groups were obtained using the TruSeq PE Cluster Kit v4-cBot-HS Illumina sequencing platform, generating a total of 1.83Gb raw reads (Table 1). After sequencing quality control, a total of 0.92Gb clean reads were obtained, with the percentage of Q30 bases ranging from 94.09 to 95.68%. All clean reads were then mapped to the Asian yellow pond turtle genome, and the match efficiency ranged from 88.18 to 90.82% (Table 1). After conducting variable splicing prediction and gene structure optimisation analysis, we identified 21,968 new genes. Out of these, 7,344 were functionally annotated.

**Table 1:** Summary of sequencing data

Sample	Total Raw Reads	Total Clean Reads	Clean Reads ≥ Q30 (%)	Total Mapping (%)	Uniquely Mapping (%)	Multiple Mapping (%)
M6-1	43,481,782	21,740,891	95.10%	38,819,094 (89.28%)	37,398,472 (86.01%)	1,420,622 (3.27%)
M6-2	44,968,352	22,484,176	95.27%	40,167,792 (89.32%)	38,662,056 (85.98%)	1,505,736 (3.35%)
M12-1	43,042,014	21,521,007	95.10%	38,253,100 (88.87%)	36,860,600 (85.64%)	1,392,500 (3.24%)
M12-2	42,424,084	21,212,042	95.27%	37,886,778 (89.30%)	36,454,303 (85.93%)	1,432,475 (3.38%)
M24-1	43,114,324	21,557,162	94.98%	38,020,470 (88.19%)	36,453,422 (84.55%)	1,567,048 (3.63%)
M24-2	43,816,554	21,908,277	95.04%	39,262,607 (89.61%)	37,740,284 (86.13%)	1,522,323 (3.47%)
M48-1	43,478,496	21,739,248	95.48%	38,974,386 (89.64%)	37,495,319 (86.24%)	1,479,067 (3.40%)
M48-2	43,944,060	21,972,030	95.24%	39,303,373 (89.44%)	37,840,028 (86.11%)	1,463,345 (3.33%)
M72-1	43,969,498	21,984,749	95.12%	39,137,319 (89.01%)	37,646,761 (85.62%)	1,490,558 (3.39%)
M72-2	43,916,848	21,958,424	94.99%	39,174,223 (89.20%)	37,685,789 (85.81%)	1,488,434 (3.39%)
MF6h-1	43,546,706	21,773,353	95.35%	39,103,422 (89.80%)	37,662,283 (86.49%)	1,441,139 (3.31%)
MF6h-2	106,369,018	53,184,509	95.65%	95,679,317 (89.95%)	91,918,225 (86.41%)	3,761,092 (3.54%)
MF12h-1	43,282,586	21,641,293	95.37%	38,468,541 (88.88%)	37,109,332 (85.74%)	1,359,209 (3.14%)
MF12h-2	43,353,906	21,676,953	94.09%	38,229,230 (88.18%)	36,764,922 (84.80%)	1,464,308 (3.38%)
MF24h-1	44,067,724	22,033,862	95.04%	39,013,694 (88.53%)	37,529,355 (85.16%)	1,484,339 (3.37%)
MF24h-2	62,335,764	31,167,882	95.58%	55,555,432 (89.12%)	53,361,504 (85.60%)	2,193,928 (3.52%)
MF48h-1	43,206,022	21,603,011	95.09%	38,531,483 (89.18%)	37,092,612 (85.85%)	1,438,871 (3.33%)
MF48h-2	43,646,946	21,823,473	94.85%	38,808,321 (88.91%)	37,277,147 (85.41%)	1,531,174 (3.51%)
MF72h-1	44,284,052	22,142,026	95.36%	39,277,003 (88.69%)	37,775,990 (85.30%)	1,501,013 (3.39%)
MF72h-2	46,183,586	23,091,793	95.68%	40,890,323 (88.54%)	39,237,494 (84.96%)	1,652,829 (3.58%)
F6-1	43,747,386	21,873,693	95.24%	39,228,875 (89.67%)	37,790,217 (86.38%)	1,438,658 (3.29%)
F6-2	43,134,878	21,567,439	95.03%	38,430,072 (89.09%)	37,019,513 (85.82%)	1,410,559 (3.27%)
F12-1	44,385,304	22,192,652	95.38%	39,996,282 (90.11%)	38,444,180 (86.61%)	1,552,102 (3.50%)
F12-2	44,661,764	22,330,882	95.03%	40,340,410 (90.32%)	38,799,532 (86.87%)	1,540,878 (3.45%)
F24-1	43,189,076	21,594,538	95.22%	38,347,067 (88.79%)	36,877,093 (85.39%)	1,469,974 (3.40%)
F24-2	44,463,082	22,231,541	95.07%	39,394,892 (88.60%)	37,873,134 (85.18%)	1,521,758 (3.42%)
F48-1	43,949,458	21,974,729	95.07%	39,324,956 (89.48%)	37,898,113 (86.23%)	1,426,843 (3.25%)
F48-2	45,317,286	22,658,643	95.22%	40,508,169 (89.39%)	39,072,622 (86.22%)	1,435,547 (3.17%)
F72-1	44,865,026	22,432,513	95.27%	40,082,434 (89.34%)	38,582,263 (86.00%)	1,500,171 (3.34%)
F72-2	39,504,260	19,752,130	95.11%	35,405,840 (89.63%)	34,079,638 (86.27%)	1,326,202 (3.36%)
FM6h-1	42,579,154	21,289,577	94.78%	37,992,466 (89.23%)	36,520,312 (85.77%)	1,472,154 (3.46%)
FM6h-2	45,840,416	22,920,208	95.48%	40,903,164 (89.23%)	39,331,697 (85.80%)	1,571,467 (3.43%)
FM12h-1	44,360,812	22,180,406	95.25%	40,286,783 (90.82%)	38,569,568 (86.95%)	1,717,215 (3.87%)
FM12h-2	44,086,682	22,043,341	95.17%	39,605,117 (89.83%)	38,137,620 (86.51%)	1,467,497 (3.33%)
FM24h-1	43,054,264	21,527,132	94.28%	38,531,698 (89.50%)	37,104,476 (86.18%)	1,427,222 (3.31%)
FM24h-2	43,444,540	21,722,270	94.89%	38,982,536 (89.73%)	37,614,794 (86.58%)	1,367,742 (3.15%)
FM48h-1	43,449,984	21,724,992	95.09%	39,012,802 (89.79%)	37,536,472 (86.39%)	1,476,330 (3.40%)
FM48h-2	43,061,960	21,530,980	94.93%	38,790,499 (90.08%)	37,356,348 (86.75%)	1,434,151 (3.33%)
FM72h-1	48,029,364	24,014,682	95.08%	42,940,136 (89.40%)	41,291,794 (85.97%)	1,648,342 (3.43%)
FM72h-2	41,789,042	20,894,521	94.69%	37,439,400 (89.59%)	36,044,558 (86.25%)	1,394,842 (3.34%)

### Identification and functional annotation of differentially expressed genes (DEGs)

**Fig. 2:**
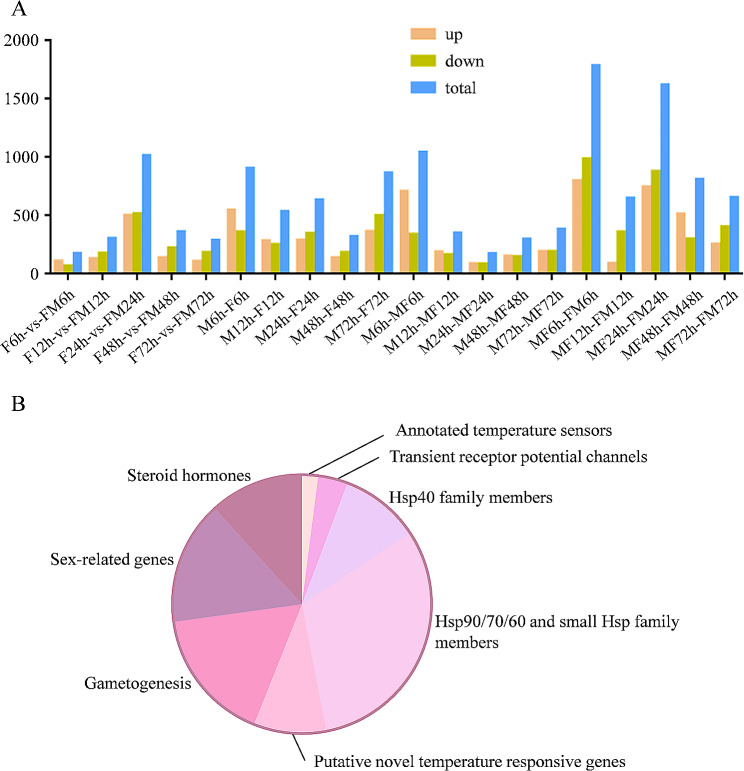
Number of DEGs in 20 comparison groups (**A**) and DEGs involved in sex determination or sex differentiation (**B**)

To compare the effect of temperature on gonadal differentiation transcript levels, we performed sex-dependent DEG analysis between MPT and FPT samples at each corresponding time point in each group. Among the 20 comparison subgroups, MF6h-vs-FM6h had the highest number of differentially expressed genes with 798 (44.78%) up-regulated and 984 (55.22%) down-regulated genes (Fig. 2A). The subgroup with the second highest number of differentially expressed genes was MF24h-vs-FM24h, with a total of 1618 DEGs. Of these, 743 (45.92%) were up-regulated and 875 (54.08%) were down-regulated. Among these DEGs, several classes of gene products that respond to temperature stimuli were identified, including the annotated temperature sensors *Calca*, *Mmp11*, transient receptor potential channels *Trpm1*, *Trpm4*, *Trpm6*, *Trpv1*, Hsp40 family members *Dnaja4*, *Dnajb1*, *Dnajb6*, *Dnajb14*, *Dnajc2*, Hsp90/70/60 and small Hsp family members *Hspa5*, *Hspa8*, *Hsp90aa1*, *Hsp90b1*, putative novel temperature-responsive genes *Eif4a2*, *Des*, *Mettl21a*, genes involved in germline development *Nanos1*, *Vasa*, sex-related genes *Runx1*, *Cirbp*, and sex steroid hormone-related genes *Esr*, *Hsd17b5*, *Hsd17b8* (Fig. 2B).

**Fig. 3:**
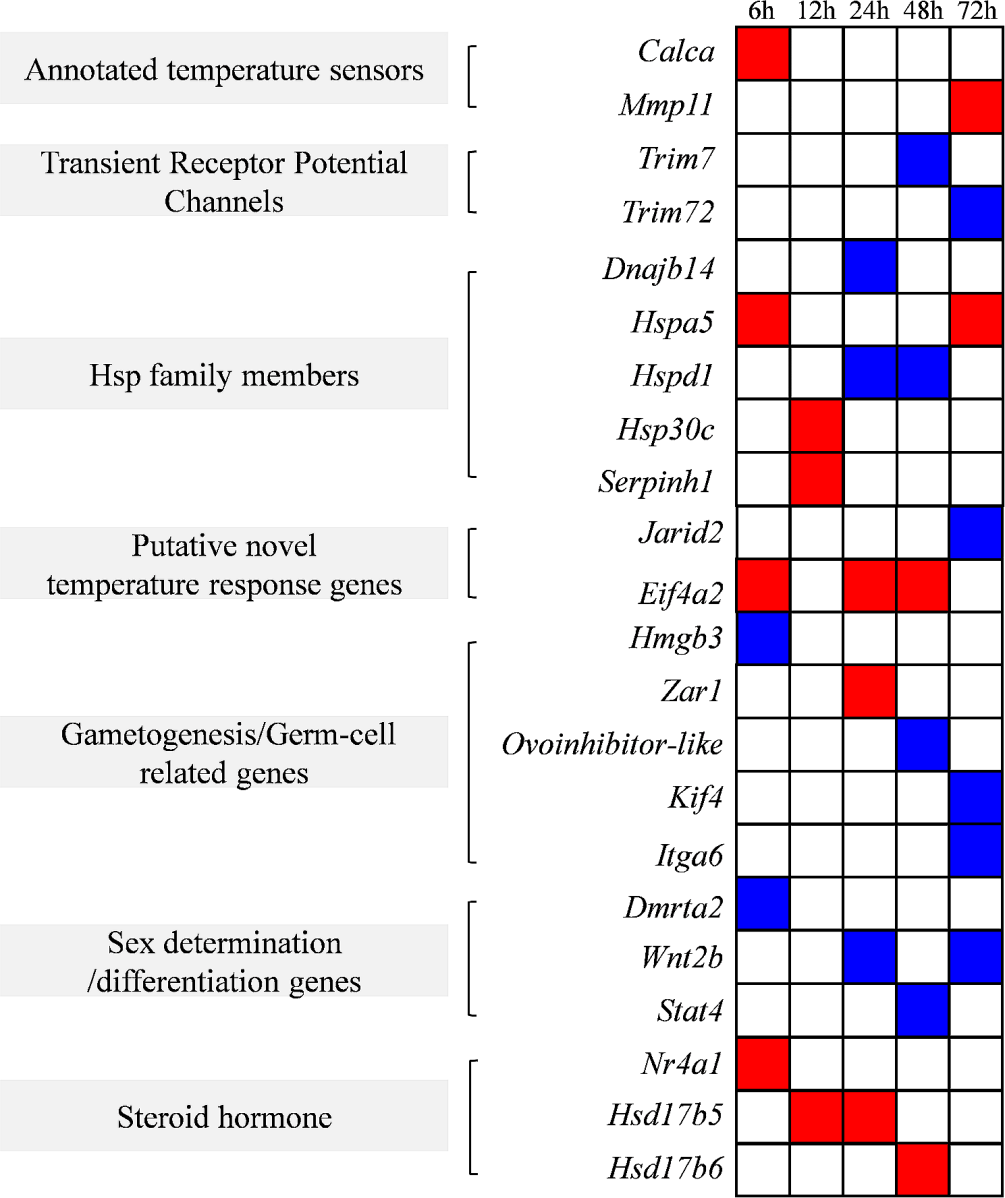
Differentially expressed genes whose transcript levels respond to MPT and FPT at stage 15, including annotated temperature sensors, transient receptor potential channels, Hsp40 family members, Hsp90/70/60 and small Hsp family members, putative novel temperature-responsive genes, genes involved in the germline, and sex steroid hormone-related genes. Blue boxes indicate expression biases in MPT embryos, and red boxes indicate expression biases in FPT embryos

The expression levels at MPT and FPT were compared at 6, 12, 24, 48 and 72 h. Six classes involved in sexual development were identified, including annotated temperature sensors (*Calca*, *Mmp11*), transient receptor potential channels (*Trim7*, *Trim72*), Hsp family members (*Dnajb14*, *Hspa5*, *Hspd1*, *Hsp30c*, *Serpinh1*), putative novel temperature response genes (*Jarid2*, *Eif4a2*), gametogenesis (*Hmgb3*, *Zar1*, *Ovoinhibitor-like*, *Kif4*), sex determination/differentiation genes (*Dmrta2*, *Wnt2b*, *Stat4*) and steroid hormone genes (*Nr4a1*, *Hsd17b5*, *Hsd17b6*, *Itga6*) were found to be differentially expressed. A total of 22 DEGs involved in gonadal sex determination were identified between the two sexes, with 10 genes being disproportionately expressed in the female gonads and 12 genes being disproportionately expressed in the male gonads (Fig. 3).

### Identification of genes with sex‑biased expression during shifts between female- and male-producing temperatures

**Fig. 4:**
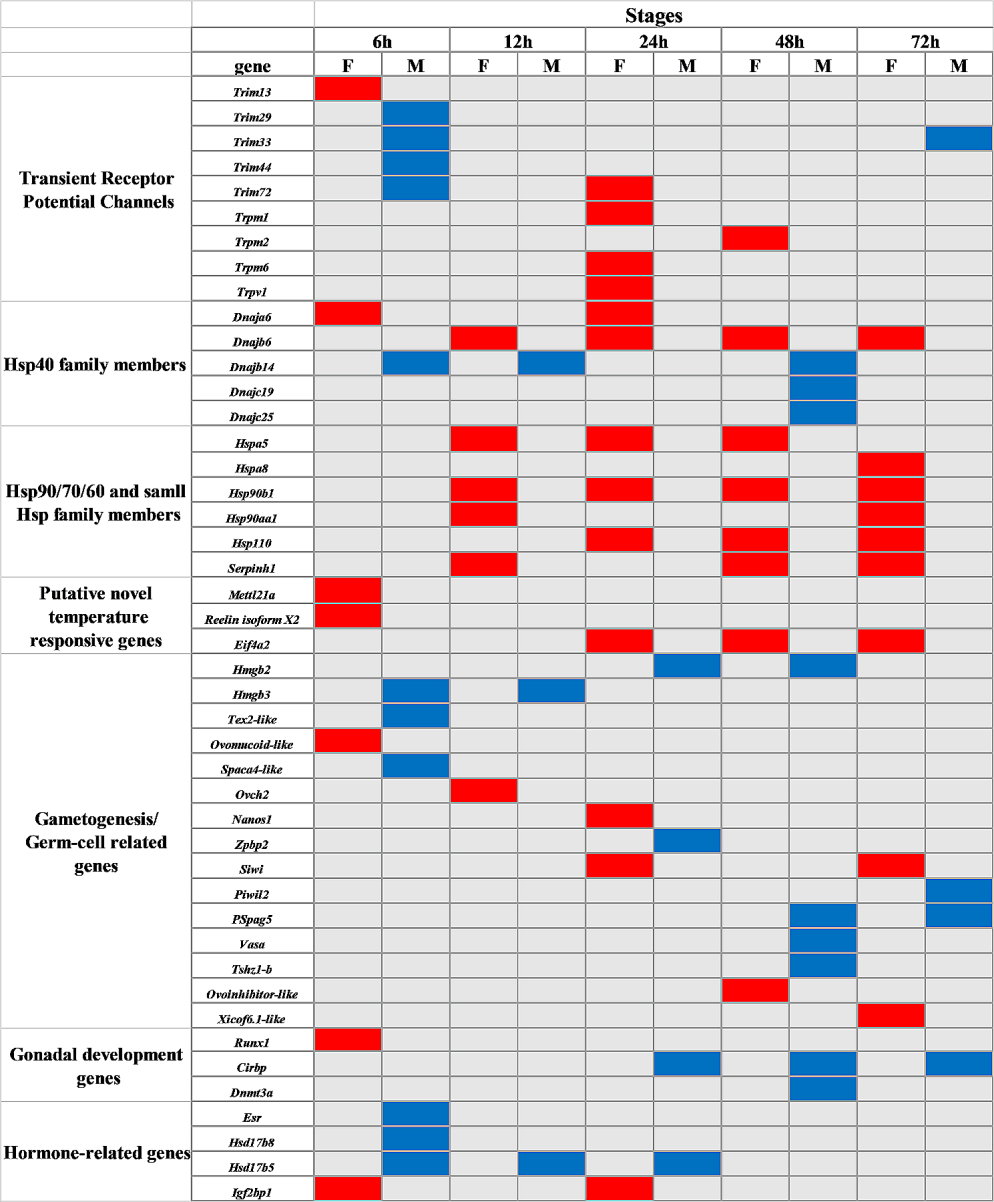
Differentially expressed genes of interest present at shifted temperatures (25 °C to 32 °C and 32 °C to 25 °C) in the thermosensitive period. Blue and red boxes indicate up- and down-regulated genes, respectively

The DEGs were screened at 6, 12, 24, 48 and 72 h after transferring eggs between female and male producing temperatures (i.e., eggs transferred from male producing temperature to female producing temperature compared to eggs transferred from female producing temperature to male producing temperature, MF-vs-FM). Similar to the sexually dimorphic genes between MPT and FPT, the analysis detected 45 specific DEGs in seven classes. Of these, 21 were upregulated (including *Trim29*, *Dnajb14*, *Hmgb2*, etc.) and 24 were downregulated (including *Dnaja6*, *Hspa5*, *Eif4a2*, etc.). These DEGs are likely candidate genes for the immediate temperature response (Fig. 4). Three genes (*Dnajb14*, *Cirbp*, *Hsp17b5*) were found to be sexually dimorphic at three time points among the upregulated DEGs, while four genes (*Trim33*, *Hmgb2*, *Hmgb3*, *Pspag5*) were differentially expressed at two time points. Among the downregulated DEGs, *Dnajb6* and *Hsp90b1* showed expression changes at four time points, while four genes (*Hspa5*, *Hsp110*, *Serpinh1*, *Eif4a2*) were skewed at three time points and four genes (*Dnaja6*, *Hsp90aa1*, *Siwi*, *igf2bp1*) were enriched at two time points during the FPT to MPT shift (Fig. 4).

**Fig. 5:**
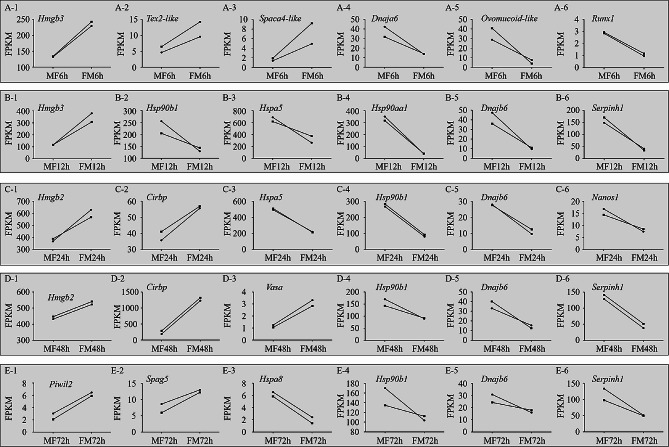
Expression level of interested DEGs at shifted temperatures (from 25 °C to 32 °C and from 32 °C to 25 °C) in the thermosensitive period. **A-1** to **A-6**, interested DEGs at 6 h of temperature shift (MF6h vs. FM6h); **B-1** to **B-6**, interested DEGs at 12 h of temperature shift (MF12h vs. FM12h); **C-1** to **C-6**, interested DEGs at 24 h of temperature shift (MF6h vs. FM6h); **D-1** to **D-6**, interested DEGs at 48 h of temperature shift (MF6h vs. FM6h); E-1 to E-6, interested DEGs at 72 h of temperature shift (MF6h vs. FM6h)

The amplitude of variation in sexually dimorphic gene expression profiles was then examined in detail, revealing several notable gene expression patterns. Following a 6-hour temperature shift (MF-vs-FM), the expression levels of *Hmgb3* (Fig. 5A-1), *Tex2-like* (Fig. 5A-2), *Spaca4-like* (Fig. 5A-3) were upregulated and displayed a male-biased expression pattern. Conversely, *Dnaja6* (Fig. 5A-4), *ovoinhibitor-like* (Fig. 5A-5) and *Runx1* (Fig. 5A-6) were enriched at the male-to-female production shift temperature and exhibited downregulated expression levels. At 12 h of temperature shift (MF-vs-FM), *Hmgb3* was significant upregulated, with a greater degree of up-regulation than at 6 h of temperature shift (Fig. 5B-1). Additionally, the relative expression of *Hsp90b1* (Fig. 5B-2), *Hspa5* (Fig. 5B-3), *Hsp90aa1* (Fig. 5B-4), *Dnajb6* (Fig. 5B-5), *Serpinh1* (Fig. 5B-6) was significantly downregulated. *Hspa5* remained downregulated at a later time point of 24 h of temperature shift (MF-vs-FM) (Fig. 5C-3). *Hsp90b1* and *Dnajb6* were downregulated at 24 (Fig. 5C-4, Fig. 5C-5), 48 (Fig. 5D-4, Fig. 5D-5) and 72 h (5E-4, Fig. 5E-5) of temperature shift. *Serpinh1* was also downregulated after 48 (Fig. 5D-6) and 72 h (Fig. 5E-6) of temperature shift. The novel candidate TSD gene for temperature-dependent sex determination in the common snapping turtle [33], *Cirbp*, was up-regulated after 24 and 48 h of temperature shift (Fig. 5C-2, Fig. 5D-2). Furthermore, several genes related to germ cell development, including *Nanos1* (Fig. 5C-6), *Vasa* (Fig. 5D-3), *Piwil2* (Fig. 5E-1) and *Spag5* (Fig. 5E-2), exhibited differential expression, indicating their sensitivity to temperature fluctuations.

## Discussion

The Asian yellow pond turtle is a large freshwater aquaculture animal with distinct sexual anamorphism, with males growing faster than females. Research into the mechanisms of its sex determination is essential for generating sex ratios and understanding the dynamics and evolutionary potential of the population [34]. Previous research has shown that the sex of the Asian yellow pond turtle is determined mainly by temperature rather than genetic factors. High incubation temperatures increase the proportion of females [25]. Therefore, it is scientifically important to investigate the genetic links between specific temperature-responsive genes and sex to determine the sexual fate of TSD turtles. The study focused on transcriptome analyses of adult gonads or embryos at different developmental stages in the Asian yellow pond turtles [18, 35], a complementary embryonic transcriptome at five time points in the early stages of gonadal differentiation after transplantation temperatures (FPT to MPT and MPT to FPT) will be able to identify potential upstream temperature-responsive genes in the male and female sex determination cascades. Here, we identified a cohort of genes involved in temperature sensing and response that affect TSD (Fig. 2). We screened for fewer sex-related genes at this early stage of gonadal development compared to later stages and adults, which is consistent with findings in other turtle species [36] or fish [37].

Candidate temperature sensors or transducers known to activate TSD in male and female gonadogenesis include genes involved in gonadal and germ-line differentiation, kinases, gametogenesis, hormone related genes, and genes linked to sex chromosomes, heat-shock genes, transient receptor potential channel genes, and histone-related genes [38, 39]. This study explores the categories of functional genes that induce temperature signalling for gonadal development. We found a range of up- and down-regulated genes, with the largest proportion being heat-shock protein (41.28%), followed by gametogenesis (16.78%) and sex differentiation-related genes (15.44%) (Fig. 2B). During stage 15 of MPT and FPT, we identified transcriptional profiles of embryonic genes that have not been previously studied in mammals, but are present in red-eared slider turtles and western painted turtles. These genes include those implicated in gametogenesis (*Hmgb3*, *Zar1*, *Ovoinhibitor-like*, *Kif4*), germ-cell related genes (*Itga6*), and genes important for sex determination/differentiation (*Dmrta2*, *Wnt2b*, *Stat4*) (Fig. 3). Genes involved in steroid hormone biosynthesis have been identified as key factors in regulating sex differentiation in turtles and fish [40]. Interestingly, we screened for DEGs including *Nr4a1*, *Hsd17b5*, *Hsd17b6* and *Itga6*. *Nr4a1* is a nuclear receptor and a member of the steroid/thyroid receptor superfamily. It has been shown to be involved in the transcriptional regulation of several steroidogenic enzyme genes in the gonads and adrenals of mammals. *Nr4a1* may affect the ability of ovarian theca cells to produce androgens by regulating the transcription of the steroidogenic enzymes StAR, Cyp11a1, Cyp17 and HSD3B2 [41]. The *Hsd17b* gene comprises two genes, *Hsd17b5* and *Hsd17b6*, both of which encode key enzymes that facilitate the conversion of androstenedione to testosterone [42]. However, we did not find differential expression of *Cyp17*, which we speculate may be due to the fact that the samples collected were at an early stage of development.

The dataset also identified genes that are sensitive to heat and showed changes in expression levels with temperature variations at five different time points after transplantation. The largest number of DEGs were found at 6 and 12 h after the transient temperature change (Fig. 2). For DEGs at 6 h, the Hsp40 family member *Dnaja6*, the gonadal development gene *Runx1*, and the hormone synthesis gene *Igf2bp1* (Fig. 4) are the earliest to respond to temperature. These genes may be important candidates for thermosensitive gonadal differentiation in TSD turtles and warrant further functional studies. Furthermore, it is worth noting that *Dnajb6* and *Hsp90b1* were downregulated at four time points (12, 24, 48 and 72 h) (Figs. 4 and 5). These genes were differentially expressed in male and female embryos as early as stage 12 in red-eared slider turtles [38] and Asian yellow pond turtles [18], but at relatively later stages in mice [43, 44]. This suggests a certain level of conservatism of these thermosensitive genes in turtles with TSD. The study shows that genes that are crucial for later stages of gonad formation in mammals have been utilized in turtles for temperature-specific responses earlier than anticipated. This highlights the significant ontogenetic evolution of the transcriptional patterns of this regulatory network in vertebrates, particularly in turtles [45]. Our analyses revealed upregulated expression of *Cirbp* at the 24 and 48-hour time points. *Cirbp* was previously found to be involve in determining gonad fate in the snapping turtle, *C. serpentina*, and is a new candidate gene for TSD turtle [33]. Although *Cirbp* is considered an upstream gene of *Foxl2*, its expression was found to be higher in males than in females in this study, suggesting that genes regulating gonadal development may also vary in turtles, which could reveal new functions for these genes in turtles.

In addition to the aforementioned candidates, several germ cell-related genes, including *Vasa*, *Nanos1* and *Piwil2*, were identified as DEGs. *Vasa* is primarily expressed in germ cells and is highly conserved across most studied species. It regulates germ cell development during embryogenesis and germ cell differentiation during gametogenesis [46]. The *Vasa* gene is first detected in Asian yellow pond turtle embryos at stage 16 and shows differential expression between male and female embryos, which providing new insights into the conservation and divergence of germ cell genes across different phyla [24]. *Nanos1* is a conserved gene that plays a crucial role in germline cell specification and differentiation [47, 48]. It also exhibits significant differences in the gonads of both sexes, indicating its importance in gonadal differentiation and development [49, 50]. *Piwil2* is believed to have essential functions in embryonic and gonadal development in *Scophthalmus maximus* and may have distinct roles in gonadal development in each sex [51]. Future studies should investigate whether these germline genes play a role in sex determination or gonadal development in the Asian yellow pond turtle.

## Conclusions

Comparative transcriptome analysis was used to identify candidates that may control sex determination and differentiation. Several known gonadal regulators and turtle-specific novel transcripts that are active well before the onset of the thermosensitive period were uncovered. One or more of these candidates may act as initial environmental response sensors, transmitting or translating temperature information to downstream testicular and/or ovarian differentiation cascades. Our work enhances the genetic resources for sex control in TSD turtles, enabling further research in this field. However, this study does not allow for the deduction of the biological functions of these genes. Further studies are necessary to identify the master genes from the earliest stage of our time course and the downstream cascade of gene expression for both male and female pathways.

### Electronic supplementary material

Below is the link to the electronic supplementary material.

Supplementary Material 1

## Data Availability

The data that support the results of this present study are available from the corresponding author upon reasonable request. The reference genome of Asian yellow pond turtle is available in the NCBI Sequence Read Archive database under the BioProject accession number SRR14883730 (BioProject ID: PRJNA740058).
