# Supplementary material for: Screening of temperature-responsive signalling molecules during sex differentiation in Asian yellow pond turtle (Mauremys mutica)
Source: BMC Genomics. 2024 Apr 18;25:383. doi: 10.1186/s12864-024-10275-5 (PMC11025153; doi:10.1186/s12864-024-10275-5)
Supplement: Supplementary file 1 — Supplementary Material 1 [file 12864_2024_10275_MOESM1_ESM.doc]

**Supplemental Information for:**

Screening of temperature-responsive signalling molecules during sex differentiation in Asian yellow pond turtle (*Mauremys mutica*)

Xiaoli Liu ^1, 2^, Haoyang Xu^1, 2^, Mingwei Peng^1, 3^, Chenyao Zhou^1, 3^, Chengqing Wei^1^, Xiaoyou Hong^1^, Wei Li^1, 2^, Chen Chen^1^, Liqin Ji^1^, Xinping Zhu^1, 2, 3*^

1. Key Laboratory of Tropical and Subtropical Fishery Resources Application and Cultivation, Ministry of Agriculture and Rural Affairs, Pearl River Fisheries Research Institute, Chinese Academy of Fishery Sciences, Guangzhou, 510380

2. College of Life Science and Fisheries, Shanghai Ocean University, Shanghai, China, 201306

3. Zhejiang Ocean University, School of Fishery, Zhoushan, 316000

Liu et al., Supplemental Figure


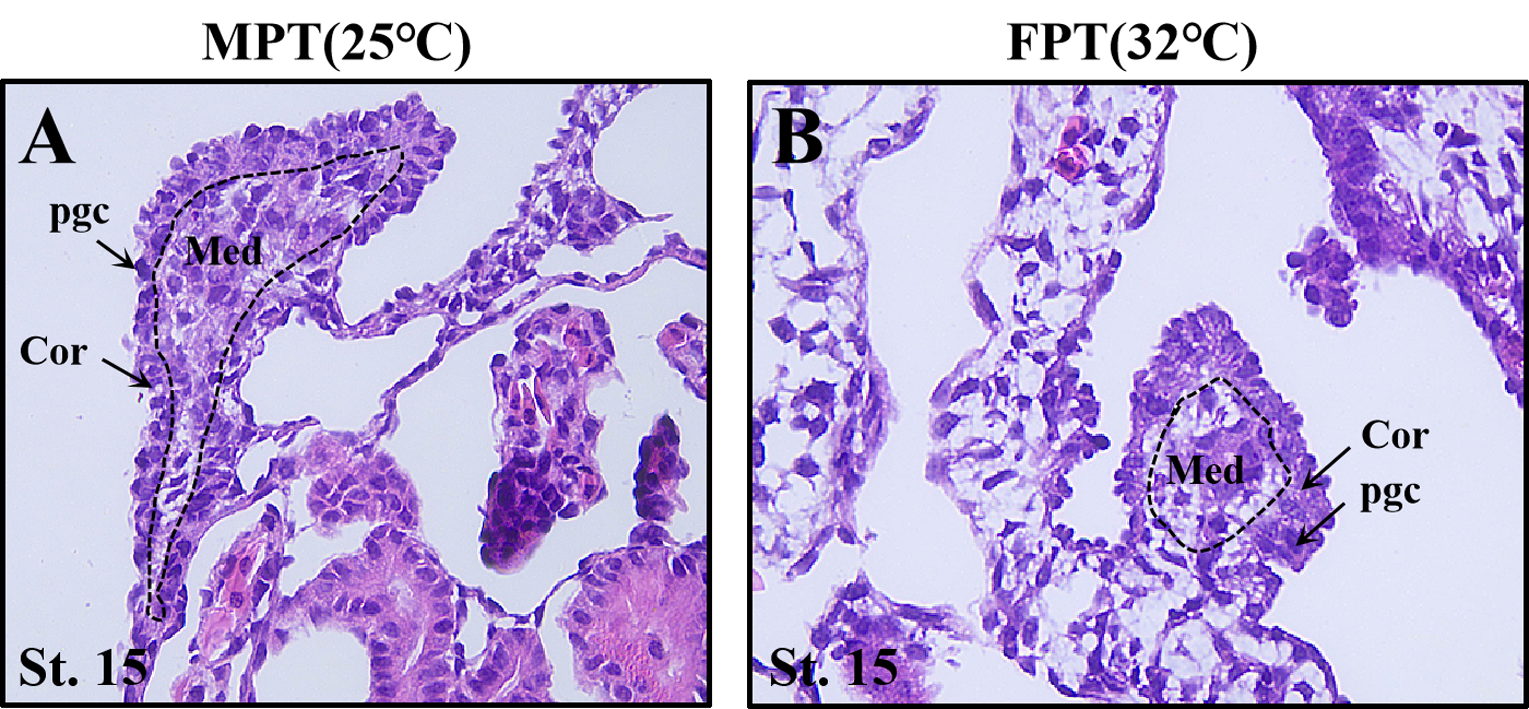


**Supplementary Figure S1**

**Supplemental Figure S1.** Hematoxylin and Eosin staining of gonadal sections from MPT (A) and FPT embryos at stage15 (B). The dashed black line indicates the border between medulla and cortex. pgc, primordial germ cells; Cor, cortex; Med, medulla.


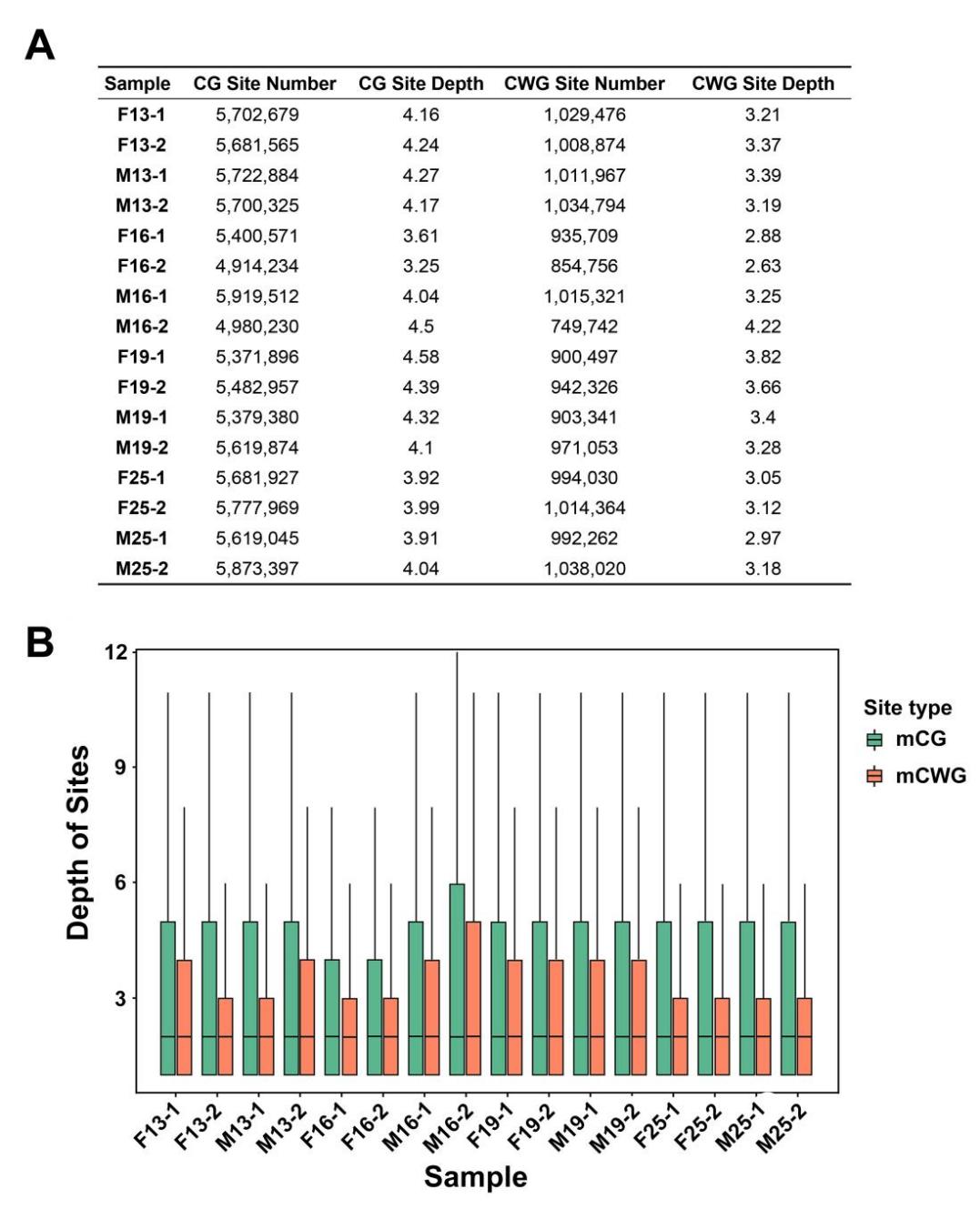


**Supplementary Figure S2**

**Supplemental Figure S2.** Statistical analysis of methylation sites. A: Depth of mCG and mCWG methylation site coverage statistics. B: Box-whisker plot of the depth distribution of mCG and mCWG.


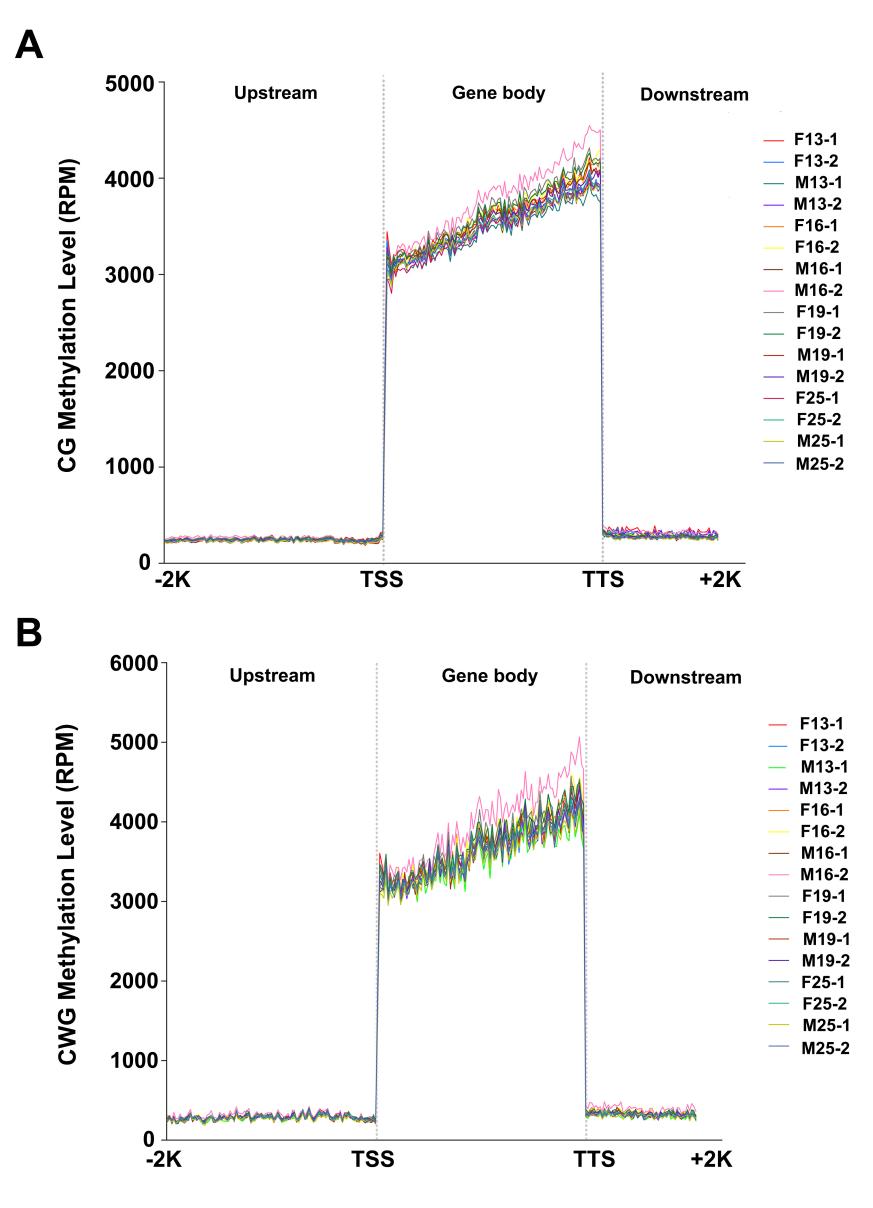


**Supplementary Figure S3**

**Supplemental Figure S3.** Methylation distribution of RefSeq genes and 2 kb flanking sequences in embryonic gonads at different stages. A: CG methylation levels in gene regions; B: CWG methylation levels in gene regions. TSS: transcription start site, TTS: transcription termination site. RPM: Reads per million mapped reads.


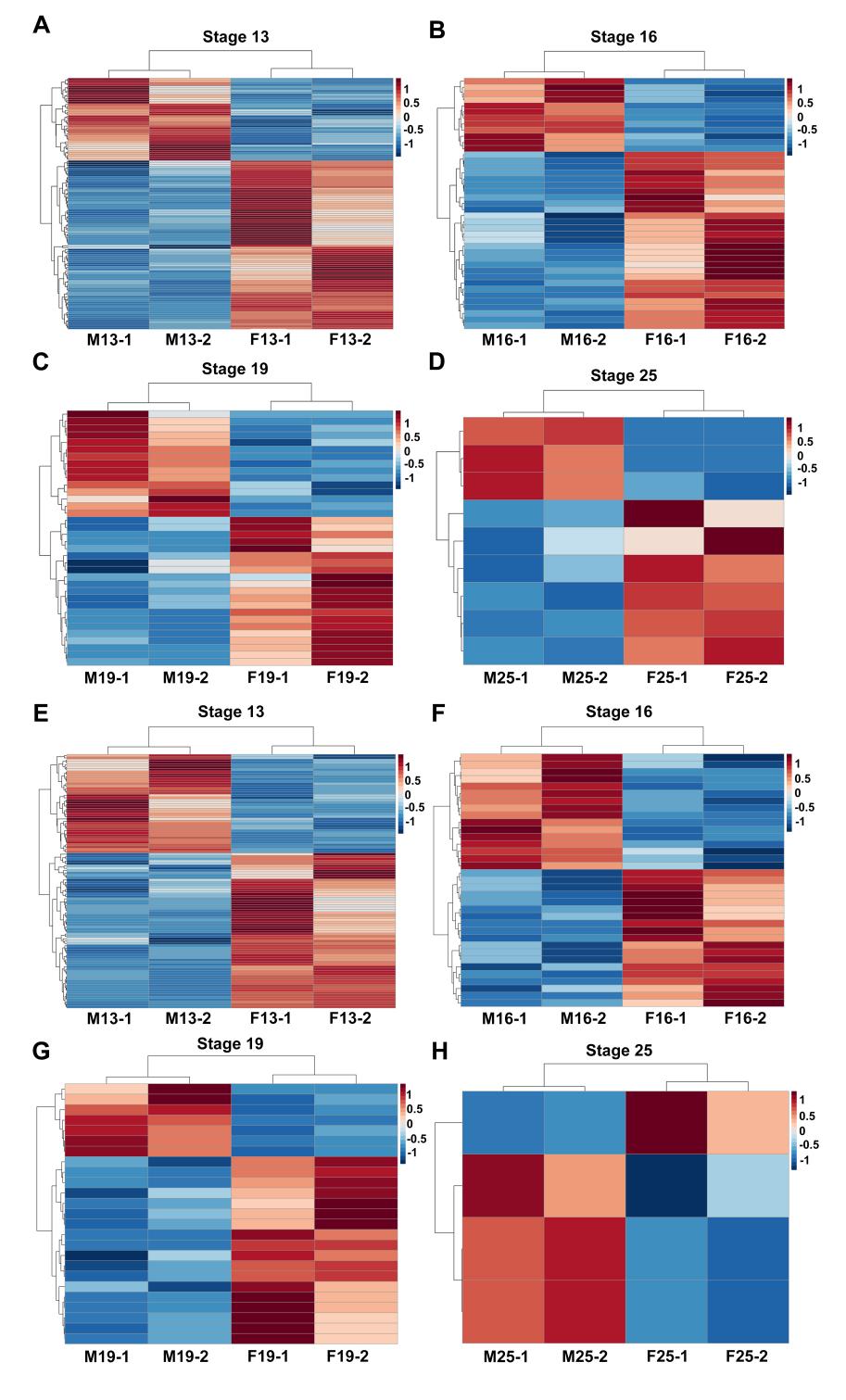


**Supplementary Figure S4**

**Supplemental Figure S4.** Heatmap illustrating differentially CG (A-D) and CWG (E-H) methylated genes in developing gonads of different stages between MPT and FPT embryos. The x-axis represents the sample, and the y-axis represents the gene. Red: higher methylation level of the differentially genes; Blue: lower methylation level of the differentially expressed genes.
